# What about spiritual needs? Care robotics and spiritual care

**DOI:** 10.3389/frobt.2024.1455133

**Published:** 2024-12-17

**Authors:** Jonas Simmerlein, Max Tretter

**Affiliations:** ^1^ Institute of Practical Theology and Psychology of Religion, University of Vienna, Vienna, Austria; ^2^ Chair of Systematic Theology (Ethics), Seminar of Systematic Theology, Friedrich-Alexander Universität Erlangen-Nürnberg, Erlangen, Germany

**Keywords:** social robots, total care, health care, wellbeing, religion, theology, ethics

## Abstract

Health is a multidimensional phenomenon encompassing physical, mental, social, and spiritual aspects. Therefore, it is only logical that good care should be holistic and include all these dimensions. However, when examining the current field of health and care robotics, the spiritual aspect is notably neglected. As a result, current health and care robots cannot provide holistic care. This paper argues that this neglect should be addressed, and, drawing on the emerging field of spiritual robotics, that spiritual aspects should receive greater attention when considering, developing, or deploying health and care robots. We also propose guidelines for equipping health and care robots with the necessary spiritual capabilities.

## 1 Introduction

Health is more than just physical wellbeing. A person with no physical ailments might still suffer with their mental health, loneliness, or an inner sense of emptiness. That’s why health should be approached holistically, taking into account not only physical aspects but also mental, social, and spiritual dimensions (Cobb et al., 2012; Oman, 2018; Timmins and Caldeira, 2019). Yet, if health is a holistic phenomenon and personal wellbeing depends on all these various factors, then care should also be understood holistically (Jasemi et al., 2017; Nauer, 2015). In fact, the holistic nature of care is increasingly recognized, as, for instance, evidenced by the principle of “total care,” which is gaining traction in nursing sciences and related fields and posits that quality care integrates physical, mental, social, and spiritual aspects (Ott et al., 2023; Radbruch and Payne, 2009).

Given the increasing involvement of robots in caregiving (Huang et al., 2023), the question arises: why do current robots employed in diverse health and care settings for a variety of purposes not provide spiritual care? While there are various robots in the health and care sector that perform physical and mental care tasks or support human caregivers, and robots’ abilities to serve as social companions are continuously being developed (Cifuentes et al., 2020), the spiritual aspect of care is largely overlooked in current health and care robotics. Therefore, in this Perspective we will focus on the spiritual aspect of care, arguing that it should not be neglected when considering, developing, or deploying what we call “health and care robots,” i.e., robots intended for use in various health and care contexts for a range of health and care.

To support our claim, we draw on our expertise as Protestant theologians and ethicists with extensive academic experience in the fields of medical ethics and caregiving, engaging with existing empirical studies to derive well-founded conclusions. Our analysis begins with an emphasis on spiritual care, illustrating its critical role in overall healthcare. We will then show that spirituality is a highly neglected aspect in today’s health and care robots. However, as we will illustrate through the broad field of spiritual robotics and several recent research projects, this neglect is not due to an inherent inability of robots to engage in spiritual tasks or a general disinterest in this area. Instead, we propose that this neglect might stem from several key concerns. Based on this, we will conclude by outlining several guidelines that may serve as a guide for equipping robots to provide spiritual care in a way that takes these concerns seriously and sensitively addresses them. Last but not least, although both authors have a theological background and emphasize the importance of theological perspectives in discussions of this topic (Tretter, 2024), we stress that our conclusions in this Perspective are not limited to theological perspectives. Instead, we present our arguments in a way that is accessible and relevant to a diverse audience.

## 2 Spiritual care as an essential aspect of healthcare

To understand the role of spiritual care in overall healthcare, it is crucial to first define what spirituality entails. While spirituality shares certain similarities with religiosity, the two concepts remain fundamentally distinct. *Religiosity*, on the one hand, is typically used to refer to the beliefs, practices, and rituals associated with established religious institutions or traditions, while *spirituality*, on the other, although there is no definition of it universally agreed on (Koenig et al., 2023), usually refers to personal beliefs, values, or practices that provide meaning and purpose in life and are often cultivated outside the framework of organized religion (Cobb et al., 2012; Koenig et al., 2023; Oman, 2018; Timmins and Caldeira, 2019). While spirituality, such as an individual’s sense of connection to something greater, was long predominantly expressed within the forms of established religion, it has become more and more distinct with the decline of organized religion. In this sense, spirituality can be considered a relatively modern phenomenon (Dahlgrün, 2009).

Similarly, spiritual care is also a modern phenomenon (Roser, 2017). In the past, the spiritual needs of patients in healthcare settings were naturally addressed by the prevailing religiosity of patients and caregivers, or through on-site religious services. However, with the decline of organized religion in many parts of society and the increasing diversification and hyper-specialization of the medical field over the last century, with specialized professionals addressing specific aspects of a patient’s health, the spiritual dimension of care has been increasingly overlooked. It was not until the 1970s that pioneers of the hospice and terminal care movement like Cicely Saunders realized that apart from physical and psychological pain, which healthcare institutions focus on, patients were suffering from social and spiritual distress as well (Nauer, 2015). The gradual increase in awareness for spiritual needs has been a rather recent phenomenon in the scientific discourse (Wang et al., 2022).

Spiritual needs include among other things prayer and religious rituals (communion, anointing, blessings, meditation, confession, chants, mantras, etc.), theological discussions about topics like life after death, forgiveness, and guilt, as well as biographical closure and remembrance. Spiritual caregivers are typically trained professionals who may either be rooted in a specific religious tradition or work independently of religious institutions. Their role is to accompany patients through the challenges of illness and the process of dying. In addition to the more obvious spiritual–and also religious–needs they accommodate, they oftentimes provide care that could also be characterized as social care: they show simple human presence and attention to patients, where the overburdened healthcare systems fail to do so, they support patients with empathy and consolation, especially when no relatives are there to do so, they function as neutral advocates between interests of the patient, the family and the professionals (Ho et al., 2017; Roser, 2017). Especially with terminally ill patients, recent research has demonstrated the value of holistic approaches to terminal care, which include the specific needs spiritual care provides (Swinton et al., 2016).

## 3 The neglect of spiritual aspects in current health and care robots

As mentioned above, the field of robotics for health and care purposes has seen significant advancements in the past years, both in research and practical application. On the research and experimental front, the development of robots for the health and care sector is progressing rapidly. New robots and robotic applications are continuously being introduced, designed to undertake various health and care tasks, with their functionalities being experimentally tested in smaller settings. On the application side, the use of robots in the health and care sector is steadily increasing. While it is not yet possible to speak of a widespread deployment of robots in health and care institutions, and despite continuing criticism questioning the purpose and effectiveness of using robots for such tasks (Wright, 2023a; 2023b), an increasing number of clinics and homes for the elderly or individuals requiring special care are already employing robots to handle specific health and care tasks.

There is a wide range of robots designed for various physical, mental, and social care tasks. *Assistive robots*, for instance, are designed to perform mechanical tasks to relieve both patients and caregivers (Ohneberg et al., 2023; Santhanaraj et al., 2021). These tasks might include safely transporting materials like bed linens around a facility (*TUG*), assisting elderly individuals with household tasks and mobility (*Toyota’s Human Support Robot*), or, in cases that have so far only been tested in prototypes and have yet to see widespread use, lifting patients from bed to wheelchair (*Robear* and *Elevon*) (Graf, 2020; Wright, 2023a). *Social robots*, often with human-like appearances, are designed to interact with people. *Pepper* and *NAO*, for example, are frequently used in nursing homes and clinics to engage residents and patients through speech recognition, facial expressions, and gestures. They motivate individuals to move, exercise, play games, or converse, thereby supporting their physical and mental wellbeing (Blindheim et al., 2023; Dawe et al., 2019). Additionally, there are *companion robots* like *Paro*, a robotic plush toy resembling a seal, which responds with sounds and movements that indicate pleasure when petted. *Paro* is often used as a social companion in nursing settings to provide emotional support and alleviate loneliness, with studies showing that Paro can indeed produce positive psychosocial effects in those who engage with it, although these effects tend to be rather short-lived (Baisch et al., 2018; Lu et al., 2021).

These examples illustrate the diverse ways robots are currently used in health and care settings, interacting with individuals to support caregiving processes and improve individuals’ overall health and fitness. However, these examples suggest that the primary focus of health and care robots is on the physical, mental, and social aspects of wellbeing.

## 4 The broad field of spiritual robotics

These shortcomings are not due to the technical impossibility of equipping robots with the ability to offer spiritual care. On the contrary, as the growing field of spiritual robotics–sometimes also called “religious robotics” – shows, efforts to integrate robots into spiritual practices and enable them to perform or guide spiritual actions have been numerous and are increasing in both quality and quantity across various spiritual and religious traditions (Balle and Ess, 2020; Simmerlein and Tretter, 2024b; Trovato, 2020).

**FIGURE 1:**
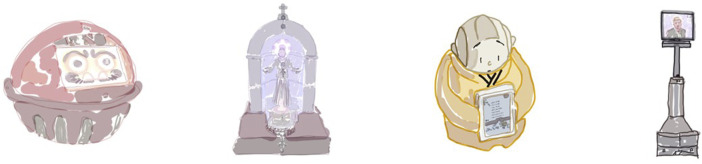
Artistic illustrations of the robots: *DarumaTO-1, SanTO, Xian’er,* and *CARL* (left to right; illustrations created by Isabella Auer).

Caring for the spiritual needs of believers and practitioners is a central concern of most religious traditions. It therefore comes as no surprise that many robotic advances in the religious sphere are aimed at satisfying those. For example, some of the robots developed by Gabriele Trovato are explicitly regarded as “religious.” *DarumaTO*, a robotic *Daruma* doll grounded in the Zen-Buddhist tradition was developed to combat loneliness of elderly Japanese citizens. The tiny round robot displays facial expressions, has speaking abilities and can be interacted with by touch. It is intended to function as a social companion to talk to and to play with (quizzes and singing). Designing it in shape of a spiritual object is supposed to make the robot more appealing to users and maybe even encourage them to open up to talk about spiritual topics (see Figure 1) (Shen et al., 2022; Trovato et al., 2019b). A similar application was built for the Catholic tradition with *SanTO*, a Catholic saint-like figure with rudimentary praying and talking abilities (see Figure 1). Both of them are capable of responding to simple inquiries with rudimentary means. They are created with the care setting in mind and tested in elder care facilities to combat loneliness and spiritual needs–e.g., having a prayer companion, talking with somebody about spiritual and religious topics–with elder citizens (Trovato et al., 2019a; Trovato et al., 2018). However, these specific applications demonstrate how far practice still lags behind well-intentioned theory. The verbal part of spiritual care like talking about existential topics is hard to satisfy by the existing robotic applications–but, looking on the rapid advances in this field in the last years, this might be tackled profoundly by more broadly implementing generative AI/chatbots.

Looking at the broader scape of what spiritual care means, other robots come into focus. Especially the ritual and accompanied meditation part has been demonstrated successfully in various cases. Take *Xian’er*, a robot monk residing in the *Longquan Monastery in Beijing* (see Figure 1). At its core this anthropomorphic robot is a chatbot algorithm that can be reached both online and in their embodied form in the monastery, where they also lead visitors in their meditation (Travagnin, 2020). *Xian’er* combines views and input of the creating monks but is also shaped by the use of visitors and their needs. It satisfies both the need for spiritual conversation to an extent as well as facilitating spiritual practice. Furthermore, the multipurpose humanoid robot *Nao* has been successfully implemented as a spiritual caregiver in care for patients with dementia. It facilitated several different muslim prayer rituals which were positively received by the patients (Ismail et al., 2018). *Pepper* has also been conducting religious practices like funerals in Japan (Cheong, 2020).

Regarding health robots as facilitators for spiritual practice and thus as contributors to spiritual care is the approach we champion in this perspective. *CARL*, a telecommunications robot utilized mainly in funeral contexts, is capable of freely moving on wheels and allows for displaying the face of its user through a video feed while simultaneously transmitting sound in both directions (see Figure 1). This enables individuals who are prevented from attending funerals due to logistical or health reasons to participate both perceptually and communicatively, thus addressing the spiritual need of closure and farewell (Arnold et al., 2021). *CARL* shows how robots extend the reach of potential therapeutic and pastoral practices. In contexts like this, robots function as an extension of the spiritual care spectrum, yet remain reliant on human input in the process. While unsupervised robots are not adequately equipped to met spiritual needs yet, aids robots can be an essential part of spiritual care.

## 5 First steps towards equipping health and care robots with spiritual capabilities

Given that there are already several robots that cater to the spiritual needs of persons, the question arises: why is this aspect of spirituality not more prominent in today’s health and care robots? This question is even more pressing given that some pioneering steps have been made in research to bridge the gap between health and care robotics and spirituality (Simmerlein and Tretter, 2024a). Notably, the European-Japanese collaborative projects *CARESSES* (2017–2020) and e-*VITA* (2021–2024) exemplify these efforts.

The goal of the *CARESSES* project was to “design the first care robots that adapt the way they behave and speak to the culture of the person they assist.” (CARESSES, 2024) Although spirituality was not a central part of the CARESSES research design and was not explicitly addressed in the project’s publications–even though some publications would have provided good opportunities to do so (Chiang et al., 2019; Sgorbissa et al., 2019; Shen et al., 2019) – the project nevertheless laid important groundwork. Given that spirituality is deeply connected to personal identity and is a key component of culture for many people, the *CARESSES* approach could be expanded to also incorporate spiritual aspects, enabling robots to respond to and engage with individuals’ unique spiritual needs.

The *e-VITA* project took an even more explicit approach to integrating spirituality. This recently completed project focused on developing an “ICT-based virtual coaching system“ capable of detecting “subtle changes in physical, cognitive, psychological and social domains of older adult’s daily life” and providing tailored recommendations to help them remain fit, mobile, and healthy (e-VITA, 2024a). To allow for comprehensive monitoring of its users and individualized recommendations, the virtual coach *e-VITA* was designed as an interface connecting various devices, including user-related devices like smartphones and fitness trackers, environmental devices such as thermometers and air quality monitors, and home-based devices like robots or smart home devices that track users’ activities. Since e-VITA was based from the start on a multidimensional health concept that also includes spiritual aspects (Jokinen et al., 2021; McTear et al., 2023; Naccarelli et al., 2023; Stara et al., 2023), spiritual robots like *SANTO*, *DarumaTO*, and *CelesTE* were also integrated into the project (e-VITA, 2024b; Naccarelli et al., 2024; Shen et al., 2022; Trovato and Weng, 2023).

First, it is important to recognize that *e-VITA’s* main objective was to develop a virtual, not a robotic, system, meaning the project did not directly address the link between health and care robots and spiritual care–though robots were consistently part of the *e-VITA* environment, and it was often emphasized that the virtual coach would eventually be compatible with various front-end devices (Shen et al., 2022; Trovato and Weng, 2023). Second, looking back at some of the project’s key empirical studies, such as those on how robots and AI systems influence users’ fitness and health, spirituality was, if at all, only examined peripherally (Bevilacqua et al., 2022; Möller et al., 2024; Naccarelli et al., 2024). Conversely, in studies examining how older adults interact with spiritual robots (specifically: *DarumaTO-3*), aspects like usability, animacy, likability, and uncanniness took precedence, while explicit spiritual care aspects–do people feel less lonely?, do they talk to *DarumaTO-3* about their deep feelings and maybe fears?, do they use the robots to help with prayer, confession or other spiritual practices? – were not explored in detail (Shen et al., 2022). Despite these shortcomings concerning the direct link between spiritual robots and spiritual care as an aspect of overall health and care, this project nevertheless contributed to bridging the gap between these fields and compellingly demonstrated that it is technically feasible and, overall, desirable to consider and incorporate spiritual dimensions in the development of health and care robots and technologies.

## 6 Guidelines for equipping robots to provide spiritual care

Altogether these findings–that it would not only be technically feasible to design health and care robots that cater to the spiritual needs of individuals but that this would also be desirable, and that substantial research and bridge-building attempts have already been made in this direction–only reinforces the questions raised above: why is the spiritual aspect of health and care so neglected when it comes to current health and care robots? Why are not, for instance, robots like *Pepper* or *NAO*, which are already being tested to offer spiritual care in experimental settings, widely equipped to provide spiritual care in caregiving contexts, thus being able to offer more holistic care? For instance, why do not they not pray with the persons they care for, quote sacred texts, or guide them in meditation?

Rather than speculating on the reasons why health and care robots have not yet been widely equipped with the ability to provide spiritual care, it seems more productive to consider the current reservations and concerns that may hinder this development:

1. First, in the Global North, affiliation with religious communities is steadily decreasing (Boyon, 2023), suggesting that many people lack interest not only in religiosity but also in spirituality, possibly even rejecting them, and therefore do not wish to be confronted with it.
2. Second, physical and mental health are often prioritized over spirituality, which is frequently seen as a component of self-actualization rather than a vital necessity. Therefore, it makes sense that health and care robots are initially designed to provide physical and mental care.
3. Third, spirituality is a highly diverse phenomenon. Different religions have various forms of spirituality, and even within a single religion, spiritual practices can vary widely, not all of which appeal to everyone. Moreover, there are those forms of spirituality that exist entirely outside the framework of religion. Equipping health and care robots to reflect this diversity and avoid offending individuals by offering inappropriate forms of spirituality is a significant challenge.
4. Fourth, spirituality is often regarded as something inherently human. Despite ongoing debates about whether forms of artificial spirituality are possible (Dorobantu and Watts, 2023; Watts and Dorobantu, 2023), there is widespread skepticism about robots being spiritual and their capabilities to offer spiritual care.
5. Fifth, for some individuals, spirituality is very intimate and sensitive, closely tied to their personality. As a result, they might be reluctant to share their spirituality with a robot (Möller et al., 2022).

In summary, these factors–many of which are supported by empirical evidence–can lead to skepticism towards spiritual care in general and the suggestion of equipping health and care robots with spiritual capabilities in particular.

These reservations and concerns indeed highlight some challenges in equipping health and care robots with spiritual capabilities, but they do not fundamentally argue against such enhancements. Rather than seeing these points as reasons to avoid providing spiritual care through robots, they can be viewed as indications of what needs to be considered when equipping robots with the ability to provide spiritual care. From the above points, several guidelines for equipping health and care robots with spiritual capabilities can be derived:

1. First, as religious affiliation is decreasing on average and some individuals might actively reject religion and spirituality, it is crucial that health and care robots provide spiritual care in a very sensitive manner. This might mean that they only offer spiritual care to individuals who actively request it or that they make individuals aware of the possibility of spiritual care once and then leave the decision up to them. In any case, they should not offer spiritual care to anyone who has declined it and must clearly accept such rejections.
2. Second, since other aspects of care are often prioritized over spiritual care, it is important to ensure that spiritual care does not come at the expense of other care aspects. Care should always be guided by the principle of total care (Ott et al., 2023), and offerings of spiritual care should be integrated into a broader range of care services.
3. Third, given that spirituality is an extremely diverse phenomenon, care offerings should not be limited to one or a few religions or favor one or a few spiritual approaches, such as prayers or meditations. Instead, it is crucial to make sure that health and care robots are capable of offering a wide range of spiritual options so that each individual, including those who do not belong to any religion—can receive spiritual care that aligns with their spirituality (Möller et al., 2022).
4. Fourth, as spirituality is often conceived as something genuinely human and many people feel more comfortable receiving spiritual care from other people, it is essential that spiritual care robots should never replace human spiritual caregivers (Tretter, 2023). Robots that are capable of offering spiritual care should only be an additional option and there should always be human spiritual caregivers available.
5. Fifth, given that spirituality is very sensitive and private, it is important that everything that occurs within the framework of spiritual care is treated with the utmost confidentiality. This means that high standards of privacy and data safety must be applied to robotic spiritual care (Möller et al., 2022).

Where these guidelines are followed, it presents a significant opportunity to equip robots with the ability to provide spiritual care. In such scenarios, the range of care offerings provided by robots can be expanded to include spiritual aspects, resulting in more comprehensive and holistic care.

## 7 Discussion

Starting from the observation that health is a multidimensional phenomenon encompassing physical, mental, social, and spiritual aspects, this paper examined whether spiritual aspects should be considered when considering, developing, or deploying health and care robots. After briefly outlining why spiritual care is an essential component of healthcare and demonstrating that spiritual aspects are significantly neglected in current health and care robotics, it was shown that this shortcoming is not due to an inherent incompatibility between robots and spirituality. On the contrary, the field of spiritual robotics highlights many successful efforts to equip robots to address individuals’ spiritual needs, while several research projects in elder care technology have also explored ways to integrate spiritual aspects into health and care technology.

Based on five possible reservations and concerns regarding robots as spiritual caregivers–including the prioritization of physical and mental care over spiritual care, the extreme diversity of spirituality, its perception as a purely human and highly intimate experience, and the fact that some individuals entirely reject spirituality–we have developed guidelines for integrating spiritual care capabilities into current health and care robots. These guidelines can assist in equipping robots with the ability to provide spiritual care, resulting in more comprehensive and holistic care.

Yet, this conclusion does not answer all questions. On the contrary, it raises several additional questions. For instance, concerning financing (*should spiritual care provided by robots be considered an additional service for which patients must pay extra, or is it included in the overall care package provided by robots?*), quality assurance (*who should be involved in assuring the quality of spiritual care, how should this quality be tested and certified?*), or the relationship between spiritual care provided by robots and that provided by humans (*do spiritual care robots complement human-provided spiritual care, serve as an alternative, or even replace it?*). Clearly, there is still much to discuss. Nevertheless, after all is said, it should have become evident that equipping health and care robots with the ability to provide spiritual care is an idea worth pursuing.

## Data Availability

The original contributions presented in the study are included in the article/supplementary material, further inquiries can be directed to the corresponding authors.
